# Eye Acupuncture Treatment for Stroke: A Systematic Review and Meta-Analysis

**DOI:** 10.1155/2015/871327

**Published:** 2015-06-16

**Authors:** Zeng-Hua Bai, Zhi-Xing Zhang, Chun-Ri Li, Mei Wang, Meong-Ju Kim, Hui Guo, Chun-Yan Wang, Tong-Wu Xiao, Yuan Chi, Lu Ren, Zhong-Yue Gu, Ran Xu

**Affiliations:** ^1^Liaoning University of Traditional Chinese Medicine, 79 Chongshan East Road, Huanggu District, Shenyang 110847, China; ^2^Department of Alternative Medicine, Nambu University, Kwangju 506-824, Republic of Korea; ^3^Department of Gynecologic Oncology, Liaoning Cancer Hospital & Institute, Shenyang 110042, China; ^4^Dalian Medical University, Dalian 116044, China; ^5^Yangxin People Hospital, Binzhou 251800, China; ^6^Benxi City Hospital for Infectious Diseases, Benxi 117022, China

## Abstract

There were applications of eye acupuncture for stroke patients. Unfortunately, similar to many other Traditional Chinese Medicine (TCM) treatments, it lacks comprehensive evaluation and system review for its effect and safety. *Objective*. This study is a systematic review to appraise the safety and effectiveness of eye acupuncture for stroke. *Methods*. “Eye acupuncture therapy” in eleven databases was searched by randomized controlled trials and quasi-randomized controlled trials. The search activity was ended in April 2014. The data were extracted and assessed by three independent authors. Rev Man 5.0 software was used for data analysis with effect estimate presented as relative risk (RR) and mean difference (MD) with a 95% confidence interval. *Results*. Sixteen trials (1120 patients) were involved with generally poor methodological quality. The study indicated that when eye acupuncture was combined with western medicine compared to western medicine, there was a significant difference in the areas of mental state, swallow function, and NDS. When eye acupuncture was combined with western medicine and rehabilitation compared to western medicine and rehabilitation, there was significant difference in the changes of SSS, FMA, and constipation symptoms evaluation. No adverse events or side effects have been reported. *Conclusions*. The current evidence is insufficient and the rigorously designed trials are warranted.

## 1. Introduction

Stroke is a neurological deficit that attributed to an acute focal central nervous system damage caused by vascular problems, such as cerebral infarction, intracerebral hemorrhage, and subarachnoid hemorrhage. It is a major cause of disability and death worldwide [1]. The burden of ischaemic and haemorrhagic stroke have increased between 1990 and 2010 in terms of the absolute number of people with incident ischaemic and haemorrhagic stroke (37% and 47% increase, resp.), number of deaths (21% and 20% increase), and Disability Adjusted of Life Years (DALYs) lost (18% and 14% increase) [2]. Eye acupuncture is a specialized and clinic approved acupuncture treatment. It was invented by Doctor Jing-Shan Peng, the professor of Liao Ning University of Traditional Chinese Medicine, in the early 1970s.

The idea of eye acupuncture was inspired by TCM theory. In his eye acupuncture theory, for the purpose of both diagnosis and treatment of disease, Dr. Peng divided the eye into four regions, eight areas, and thirteen points [3]. Eye acupuncture therapy is thought to be a kind of microacupuncture because it is believed that the stimulations of the eye around the orbital margin can open the meridians, invigorate blood, stop pain, calm the “Shen,” and regulate “Zang Fu” function [4].

Standardized manipulation of eye acupuncture is various [5]. It could be the vertical insertion within the orbital cavity, horizontal insertion outside the orbital cavity, pricking acupuncture, double insertion, and successive insertion within and outside the orbital cavity.

Since it was invented, the eye acupuncture has been practiced in Liaoning University of Traditional Medicine for more than 40 years. Thousands of stroke patients received this special treatment. Eye acupuncture has produced a tremendous clinical significance. Today, eye acupuncture is widely used in clinical treatment including: cerebrovascular disease, pain, neurological disorders, and mental disease [6, 7].

There are about 400 trials related to the eye acupuncture stored in the database of China National Knowledge Internet (CNKI). It seems that there is a large data of applications of eye acupuncture treatment for stroke. The problem is that, similar to other effective TCM treatments, it still lacks comprehensive evaluation and system review. Thus, systematic review and meta-analysis of eye acupuncture treatment are necessary and will have a great significance for study in stroke related treatment and rehabilitation.

## 2. Material and Methods

### 2.1. Protocol and Registration

A protocol of this systematic review was published in “eye acupuncture therapy for stroke: a systematic review of randomized controlled trials” (http://www.crd.york.ac.uk/prospero/display_record.asp?ID=CRD42014009632#.VHqcSNJPgoE).

### 2.2. Inclusion Criteria

As interventions, randomized controlled trials (RCT) and quasi-randomized controlled trials (Quasi-RCT) of eye acupuncture were included in this study. There was no limitation on language of publication or publication type.

According to the clinical criteria of the World Health Organization (WHO 1970), patients without limitations on age or gender were included if they were diagnosed as stroke patients. Patients were confirmed by purely clinical features or by the result of computed tomography (CT) or magnetic resonance imaging (MRI). Patients with ischemic as well as hemorrhagic stroke but not subarachnoid hemorrhage or subdural hematoma were considered for inclusion in the review.

The interventions include eye acupuncture and combined treatments, such as eye acupuncture combining with western medicine treatment, herbal treatment, rehabilitation therapy, or other alternative treatments. The controls could be western medicine treatment, herbal treatment, rehabilitation therapy, or other alternative treatments. Trials would be excluded if it related to any acupuncture treatment other than eye acupuncture in order to eliminate the influence of different acupuncture methods.

### 2.3. Identification and Selection of Studies

The relevant articles in the following databases were searched: Cochrane stroke Group Trials Register, The Chinese Stroke Trials Register, The Chinese Acupuncture Trials Register, MEDLINE, EMBASE, Alternative Medicine Database, Cumulative Index to Nursing and Allied Health Literature (CINAHL), The Chinese Biological Medicine Database (Sino Med), China National Knowledge Infrastructure (CNKI), VIP Database, and Wan fang Database.

The search activity was ended in April 2014. The following search terms were included: Ischemic stroke, Cerebral infarction, Cerebral hemorrhage, Cerebrovascular accident (CVA), Eye acupuncture, Random; Chinese phrases “zhong feng,” “nao cu zhong,” “nao xue guan bing,” “ban shen bu sui,” “pian tan,” “nao geng si,” “nao geng se,” “nao chu xue,” “nao yi xue,” “nao xue shuan,” “nao shuan se,” “qiang xi xing geng si,” “yan zhen,” and “sui ji.”

### 2.4. Data Extraction and Quality Assessment

The literature searching (BZH, ZYY), study selection (ZZX, ZYY), and data extraction (BZH, ZYY) were conducted by three independent authors. The extracted data include the name of author, title of study, year of publication, study size, age and gender of the participants, outcomes, adverse effects, prick depths of eye acupuncture, and eye acupoints for each study. Disagreement was resolved by discussion, and consensus was reached through a third party (LCR).

### 2.5. Data Analysis

Rev Man 5.0 software was used for data analysis. The effect estimates were presented as relative risk (RR) and mean difference (MD) with a 95% confidence interval. If a sufficient number in randomized trials were identified, the subgroup analyses for the outcomes, such as ADL, MRS, OHS, NIHSS, CSS, MMT, HAMD, MMSE, and WST, would be carried out.

Meta-analysis could be performed if the trials had a good homogeneity on study design, participants, interventions, controls, and outcome measures. Heterogeneity [8] between studies could be investigated by *I* ^2^ statistic which quantifies inconsistency across studies. If an *I* ^2^ was larger than 50%, it could indicate the possibility of heterogeneity. Both fixed effect model and random effect model would be used if there was a possibility of statistical heterogeneity among trials. The fixed effect model would be used for meta-analysis, if *I* ^2^ is less than 50%. The missing data could be obtained from the original trial authors. If a sufficient number of randomized trials were identified, the sensitivity analyses would be performed to explore the influence of trial quality for effect estimates. The adequacy of generation of allocation sequence, concealment of allocation, doubles blinding, and use of intention-to-treat (yes or no) were included as the quality components of methodology.

## 3. Results

### 3.1. Description of Studies

**Figure 1:**
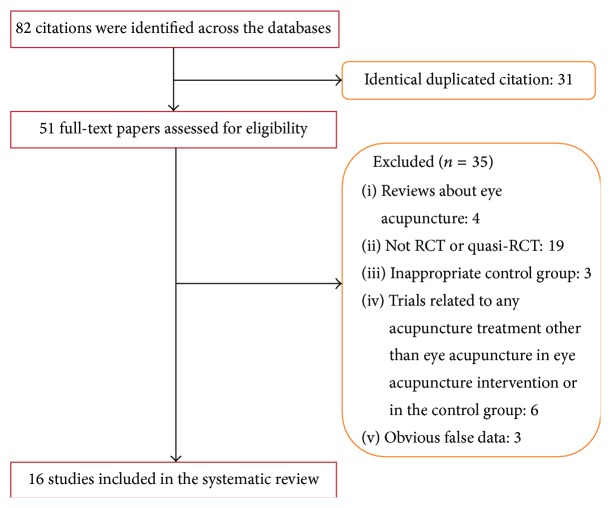
Flow chart of study selection.

16 randomized trials [9–24] were included in this review. Five trials were reported as thesis [11, 13, 14, 19, 22], and the remaining 11 trials were published in Chinese journals. A flow chart depicting the search process and study selection is shown in Figure 1. 16 RCTs and a total of 1120 stroke patients were involved in this review (69 patients per trial).

The intervention time point for ischemic stroke and hemorrhagic stroke in this study was varying from 1–3 days to more than 6 months.

The content of intervention includes eye acupuncture, eye acupuncture combined with western medicine, TCM herbal treatment, and rehabilitation. The control included western medicine, TCM herbal treatment, and rehabilitation.

The outcomes were different. As the primary outcome, CSS (Chinese Stroke Scale) was reported in 7 trials [9, 11, 13–15, 20, 22]. Activities of Daily Living (ADL) were reported in three trials [12, 17, 22]. HAMD (Hamilton Depression Scale) and WST (water swallow test) were reported, respectively, in two trials [16, 23]. The first defecation time and constipation symptoms were evaluated in one trial [19]. SSS (Scandinavian Stroke Scale) and FMA (Fugl-Meyer Scale) were assessed in one trial [17]. MMSE (Mini-Mental State Examination) was reported in one trial [18]. The ranked data for effect judgment based on clinic neurological function deficit scale (NDS) was applied in one trial [10]. As secondary results, the level change of ET and that of CGRP were reported in three trials [10, 13, 14, 24] and the level change of FIB [9] was reported for pathological improvement. The change of CRP level was observed in one trial [15]. VEGF (vascular endothelial growth factor) at the end of treatment was detected in one trial [25]. The characteristic of all included studies has been presented in Table 1.

### 3.2. Methodological Quality

**Figure 2:**
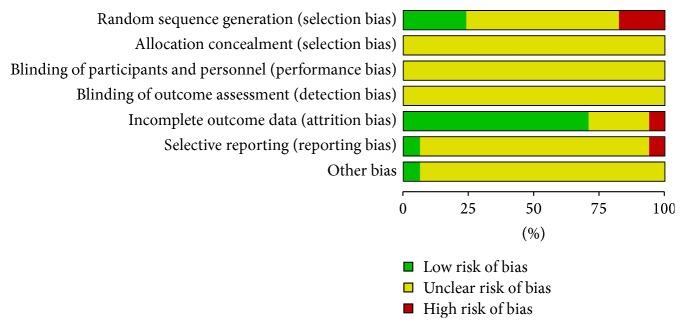
Methodological quality.

The study shows that the quality of all included trials is poor. Five trials [10, 13–15, 22] used random number table to allocate treatment. Three trials [11, 19, 21] were quasi-randomized. In these 3 trials, the patients were allocated alternately according to the visiting time point with the doctors in hospital. Nine trials did not describe the details of sequence generation. Neither adequate concealment nor blinding method was used in all trials. No follow-up document was provided. Protocols were not available. The missing data in three trials [12, 15, 25] were not available. Methodological quality has been summarized in Figure 2.

### 3.3. Effects of Interventions

Results of meta-analysis were listed in Table 2 (estimate effect of included trials in meta-analyses).

#### 3.3.1. Changes of CSS at the End of Treatment

The outcome of CSS at the end of the treatment was measured in 8 trials [9, 11, 13–15, 20, 22] with 452 patients. When eye acupuncture is combined with western medicine compared to western medicine [9, 13–15], there was an obvious difference (MD −4.24, 95% CI −5.59 to −2.89 Fixed, *I* ^2^ = 31% Fixed). One trial [11] compared the eye acupuncture combined with TCM herbal treatment to TCM herbal treatment, and there was a clear difference (MD −2.89, 95% CI −4.15 to −1.63). There was a significant difference between eye acupuncture combined with rehabilitation and western medicine versus rehabilitation with western medicine [22] (MD −2.40, 95% CI −3.76 to −1.04). There was no significant difference between eye acupuncture combined with rehabilitation and rehabilitation [20] (RR −2.40, 95% CI −4.87 to 0.07).

#### 3.3.2. Changes of ADL at the End of Treatment

The change of ADL score was measured in 3 trials [12, 17, 22] with 207 patients. Two of these trials [17, 22] were collected on continuous variable with 140 patients. The data in the other trial [12] were not available. There was a significant difference when eye acupuncture was combined with rehabilitation and western medicine versus rehabilitation and western medicine [17] (MD 17.60, 95% CI 14.19 to 21.01). One trial [22] indicated that there was a significant difference between eye acupuncture combined with western medicine treatment and western medicine treatment (MD 4.67, 95% CI 1.45 to 7.89).

#### 3.3.3. Changes of SSS at the End of Treatment

The SSS score at the end of treatment was applied in 1 trial [17] with 80 patients. There was a significant difference between acupuncture combined rehabilitation and western medicine versus rehabilitation and western medicine (MD 12.41, 95% CI, 8.92 to 15.90).

#### 3.3.4. Changes of FMA Assessment at the End of Treatment

The FMA assessment at the end of treatment was applied in 1 trial [17] with 80 patients. When eye acupuncture was combined with rehabilitation and western medicine versus rehabilitation and western medicine, there was a significant difference (MD 8.31, 95% CI, 3.15 to 13.47).

#### 3.3.5. Changes of HAMD Score at the End of Treatment

The changes of HAMD at the end of treatment were observed in 1 trial [23] with 156 patients. There was no significant difference between eye acupuncture combined with western medicine and western medicine (MD −0.82, 95% CI −1.87 to 0.23).

#### 3.3.6. Changes of MMSE at the End of Treatment

The changes of MMSE at the end of treatment were observed in 1 trial [18] with 50 patients. There was a significant difference between eye acupuncture combined with western medicine treatment and western medicine treatment (MD 1.60, 95% CI 0.28 to 2.92).

#### 3.3.7. Changes of SWT at the End of Treatment

The changes of SWT at the end of treatment were observed in 1 trial [16] with 100 patients. There was a significant difference between the eye acupuncture combined with western medicine and western medicine (RR 1.24, 95% CI 1.03 to 1.49).

#### 3.3.8. Changes of Constipation Symptoms and First Defecation Time Evaluation at the End of Treatment

The first defecation time and constipation symptoms at the end of treatment were evaluated in 1 trial [19] with 60 patients. In the comparison of eye acupuncture combined with rehabilitation and western medicine versus rehabilitation and western medicine, there was a significant difference in the constipation symptoms evaluation (MD −4.78, 95% CI −5.14 to −4.42) as well as the first defecation time (MD −1.03, 95% CI −1.46 to −0.60).

#### 3.3.9. Changes of NDS

The changes of NDS score at the end of treatment were checked in 1 trial [10] with 120 patients. There was a significant difference between eye acupuncture combined with western medicine and western medicine (RR 1.08, 95% CI 0.93 to 126).

#### 3.3.10. Changes of ET Level at the End of Treatment

The changes of ET level at the end of treatment were checked in 4 trials [10, 13, 14, 24] with 246 patients.

There was no significant difference between eye acupuncture combined with western medicine treatment and western medicine treatment in 2 trials [10, 14] (MD, 30.40, *I* ^2^ = 100%, 95% CI −43.65 to 104.46 Random). There was no significant difference between eye acupuncture combined with rehabilitation and rehabilitation in 1 trial [13] (MD −10.71, 95% CI −28.9 to 6.67). There was a significant difference between eye acupuncture and western medicine treatment in 1 trial [24] (MD, −0.64, 95% CI −1.17 to −0.12).

#### 3.3.11. Changes of CGRP Level at the End of Treatment

There was no significant difference between eye acupuncture combined with western medicine treatment and western medicine treatment (MD 1.48, 95% CI −5.31 to 8.27, *I* ^2^ = 28%, Fixed).

#### 3.3.12. Changes of FIB Level at the End of Treatment

The changes of FIB level at the end of treatment were observed in 1 trial [9] with 120 patients. There was a significant difference between eye acupuncture combined with western medicine treatment and western medicine treatment (MD −0.72, 95% CI, −1.09 to −0.35).

#### 3.3.13. Changes of CRP Level at the End of Treatment

The changes of CRP level at the end of treatment were observed in 1 trial [15] with 90 patients. There was a significant difference between eye acupuncture combined with western medicine treatment and western medicine treatment (MD −5.86, 95% CI, −7.54 to −4.18).

#### 3.3.14. Changes of VEGF Level at the End of Treatment

The changes of VEGF level at the end of treatment were observed in 1 trial [25] with 60 patients. There was no significant difference between eye acupuncture and western medicine (MD 0.02, 95% CI, −0.49 to 0.53).

Ignoring the methodological quality of included trials, the results showed some effect in independency and symptom alleviation. As eye acupuncture is combined with western medicine versus western medicine, effects appeared in the outcomes of CSS, ADL, FIB, SWT, CRP, and FIB. Eye acupuncture combined with TCM herbal treatment showed more effectiveness than TCM herbal treatment in the outcome of CSS. The outcomes of ADL have showed superiority of eye acupuncture combined with rehabilitation compared to rehabilitation. The outcomes of ADL, SSS, FMA, constipation symptoms, and first defecation time were more effective in eye acupuncture combined with rehabilitation and western medicine as compared to rehabilitation and western medicine.

### 3.4. Adverse Event

No adverse events or side effects have been reported during or after the eye acupuncture treatment according to the trials.

## 4. Discussion

The focal points in this study are the safety and effectiveness of eye acupuncture for stroke. The study demonstrated that eye acupuncture is a safe and effective treatment for stroke patients on symptoms alleviation and the dependency in the results of CSS, SSS, ADL, FMA, MMSE, HAMD, WST, and first defecation time as well as the biochemistries tests (CRP, ET, VEGF, and CGRP).

However, there were several limitations of this review. The quality of the included studies was poor because there were a mass of trials either having high or unclear risk of bias. 13% trials of random sequence generation results were of high risk and 53% trials were unclear. One trial was high risk and the rest were unclear in blinding and the same result appeared again in allocation concealment. No trials about adverse events or death were mentioned so it was impossible to get any information about safety and no trials have had the follow-up observation either. There were some therapeutic effects, but the outcomes did not focus on commonly used evaluation standards.

There were eight areas and thirteen points for eye acupuncture, but it was noticeable that the location of eye acupoints is different in trials according to the mentioned intervention methods. We wish that the locations of eye acupoints could be unified according to Standardized Manipulations of Eye Acupuncture [5]. Furthermore, trials of eye acupuncture therapy should follow the Standards for Reporting Interventions in Clinical Trials of Acupuncture (STRICTA) [26] to confirm the effect in stroke and facilitate a meta-analysis. It has been recommended that primary outcome measures for stroke should be at the level of Activities of Daily Living and the outcome should be assessed at 6 months [27].

There was no data indicating adverse events or death. But considering the position of needling, prick depths, sense of fear that patients might confront, and other potential risks for stroke, the author strongly suggests safety evaluation and psychology evaluation should be carried out for eye acupuncture.

## Figures and Tables

**Table 1:** Characteristic of all included trials.

Study ID	Study type	Sample size (T/C, male/female)	Age (yr, T/C)	Ischemic or hemorrhagic	Intervention		Duration	Area of eye acupuncture intervention	Prick depth	Outcomes
					Eye acupuncture intervention	Control intervention				
Wang et al. (2008) [9]	RCT	T: 60 (32/28) C: 60 (29/31)	T: 66 (42~70) C: 64 (41~70)	Ischemic	(1) Eye acupuncture (2) Basic treatment	Basic treatment (1) Shuxuening injection (extract of Ginkgo) (2) Citicoline Injection (3) low-dose aspirin	14 days	Major acupoints: upper jiao, lower jiao Minor acupoints: liver, kidney, spleen, and heart	NA	CSS; FIB

Zhou et al. (2011) [10]	RCT	T: 60 (46/16) C: 60 (42/18)	NA	Ischemic	(1) Eye acupuncture (2) Basic treatment	Basic treatment (1) Sodium Ozagrel injection (2) Citicoline injection (3) Low-dose aspirin	15 days	Major acupoints: upper jiao, lower jiao Minor acupoints: liver, kidney, spleen, and heart	2 mm along the cavity orbital	NDS; ET; CGRP

Liu (2010) [11]	Q-RCT	T: 28 C: 28	NA	Ischemic	(1) Eye acupuncture (2) Buyang Huanwu Decoction	Buyang Huanwu decoction	3 weeks	Major acupoints: upper jiao, lower jiao, spleen, and heart	NA	CSS

Pang (2006) [12]	RCT	T: 34 C: 34	T: (40~70) C: (40~70)	Ischemic and hemorrhagic	(1) Eye acupuncture (2) Rehabilitation training (3) Basic treatment: medicine was not available	(1) Rehabilitation training (2) Basic treatment: medicine was not available	>38 days	Major acupoints: upper jiao, lower jiao Minor acupoints: liver, kidney, and heart	9~10.5 mm in orbit	ADL

Cui (2009) [13]	RCT	T: 8 (5/3) C: 10 (6/4)	T: 51.31 ± 13.25 (40~75) C: 51.59 ± 12.89 (40~75)	Ischemic	(1) Eye acupuncture (2) Basic treatment	Basic treatment (1) Sodium Ozagrel injection (2) Citicoline injection (3) Low-dose Aspirin	2 weeks	Major acupoints: upper jiao, lower jiao Minor acupoints: liver, kidney, heart, spleen, and large intestine	3 mm along the cavity orbital	CSS; ET; CGRP

Li (2010) [14]	RCT	T: 23 (12/11) C: 25 (13/12)	T: 52.29 ± 14.89 (40~75) C: 52.46 ± 13.35 (40~75)	Ischemic	(1) Eye acupuncture (2) Basic treatment	Basic treatment (1) Sodium Ozagrel injection (2) Citicoline injection (3) Low-dose dose aspirin	2 weeks	Major acupoints: upper jiao, lower jiao Minor acupoints: liver, kidney, heart, spleen, and large intestine	2 mm along the cavity orbital	CSS; ET; CGRP

Wang et al. (2007) [15]	RCT	T: 45 (24/21) C: 45 (26/19)	T: 63.24 (40~70) C: 64.98 (43~70)	Ischemic	(1) Eye acupuncture (2) Basic treatment	Basic treatment (1) Shuxuening injection (2) Citicoline injection (3) Low-dose aspirin	14 days	Main eye acupoints: upper jiao, lower jiao Minor acupoints: liver, kidney, heart, spleen, and stomach	NA	CSS; CRP

Li and Wang (2009) [16]	RCT	T: 50 C: 50	T: (42~75) C: (42~75)	Ischemic	(1) Eye acupuncture (2) Basic treatment: medicine was not available	Basic treatment: medicine was not available	2 weeks	Major acupoints: upper jiao	7.5 mm in orbit	WST

Chen et al. (2007) [17]	RCT	T: 40 (24/16) C: 40 (22/18)	T: 68.1 ± 8.2 (40~80) C: 67.3 ± 11.1 (40~80)	Ischemic	(1) Eye acupuncture (2) Rehabilitation training based on the Bobath (3) Basic treatment	(1) Rehabilitation training based on the Bobath (2) Basic treatment (a) t-PA (b) Aspirin (c) Mannitol injection	3 months	Major acupoints: upper jiao, lower jiao. Minor acupoints: liver, gallbladder, kidney, heart, spleen, and middle jiao	NA	SSS; ADL; FMA

Li (2009) [18]	RCT	T: 25 C: 25	T: (50~75) C: (50~75)	Ischemic	(1) Eye acupuncture (2) Basic treatment: medicine was not available	Basic treatment: medicine was not available	2 weeks	Major acupoints: upper jiao, kidney, and spleen	7.5 mm in orbit	MMSE

Xi (2011) [19]	Q-RCT	T: 30 (16/14) C: 30 (18/12)	T: (35~75) C: (35~75)	Ischemic and hemorrhagic	(1) Eye acupuncture (2) Basic treatment: medicine was not available (3) Rehabilitation training: methods were not available	(1) Basic treatment: medicine was not available (2) Rehabilitation training: methods were not available	7 days	Major acupoints: lower jiao, lung, and spleen	NA	First defecation time; constipation symptoms evaluation

Jiang (2009) [20]	RCT	T: 30 C: 30	T: (40~70) C: (40~70)	Ischemic and hemorrhagic	(1) Eye acupuncture (2) Rehabilitation training	Rehabilitation training	48 days	Major acupoints: upper jiao, lower jiao, kidney, and liver	2 mm along the cavity orbital	CSS

Ren and Lin (2005) [21]	Q-RCT	T: 30 (21/9) C: 28 (20/8)	T: 54 (32~78) C: 53 (37~83)	Ischemic and hemorrhagic	(1) Eye acupuncture (2) Basic treatment	Basic treatment (1) 20% mannitol (2) Cerebrolysin (3) Huatuo Zaizao pill (4) Hemorrghagic: PAMBA Nimodipine Nifedipine (5) Ischemic: Low molecular dextran ATP Cytochrome C for injection Dicoumarin Nimodipine Aspirin	30 days	Major acupoints: upper jiao, lower jiao Minor acupoints: liver, gallbladder, kidney, and heart	2 mm along the cavity orbital	Treatment efficiency

Gao (2012) [22]	RCT	T: 30 (18/12) C: 30 (20/10)	NA	Ischemic	(1) Eye acupuncture (2) Basic treatment	Basic treatment (1) Aspirin or Clopidogrel Other medicines were not available	14 days	Major acupoints: liver, gallbladder, kidney, and heart.Minor acupoints: upper jiao, lower jiao, heart, spleen, stomach, large intestine, and bladder	2 mm along the cavity orbital	ADL; CSS

Huang (2013) [23]	RCT	T: 80 (43/37) C: 76 (40/36)	T: 61.10 ± 10.12 (40~76) C: 55.72 ± 9.02 (40~76)	Ischemic and hemorrhagic	(1) Eye acupuncture (2) Neurostan	Neurostan	8 weeks	Major acupoints: liver, middle jiao, heart Minor acupoints: kidney, spleen, and gallbladder	2 mm along the cavity orbital	HAMD

Xu et al. (2006) [24]	RCT	T: 34 (18/16) C: 26 (16/10)	T: 62.5 ± 17.5 C: 64.2 ± 7.7	Ischemic	Eye acupuncture	Basic treatment: the medicine might be Xingding injection, compound danshen injection, and Deproteinized calf blood injection	22 days	Major acupoints: upper jiao, lower jiao Minor acupoints: liver, kidney, and heart	NA	ET

Dong (2009) [25]	RCT	T: 38 (20/18) C: 34 (18/16)	T: 63.2 ± 12.5 C: 65.1 ± 8.6	Ischemic	Eye acupuncture	Basic treatment: the medicine might be Deproteinized calf blood injection, Shuxuening injection (extract of Ginkgo), Sanqi Panax Notoginseng injection	7 days	Major acupoints: upper Jiao, lower Jiao. Minor acupoints: liver, kidney, and heart	NA	VEGF

Notes: (1) ADL: Activities of Daily Living. (2) CGRP: calcitonin gene related peptide. (3) CRP: C-reactive protein. (4) CSS: Chinese Stroke Scale. (5) ET: endothelin. (6) FIB: fibrinogen. (7) FMA: Fugl-Meyer Scale. (8) HAMD: Hamilton Depression Scale. (9) MMSE: Mini-Mental State Examination. (10) SSS: Scandinavian Stroke Scale. (11) NDS: clinic neurological function deficit scale. (12) VEGF: vascular endothelial growth factor.

**Table 2:** Estimated effect sizes of included trials in meta-analyses.

Trials	Participants	Estimate effects
(1) **Changes of CSS scores for neurological assessment**		
(1.1) Eye acupuncture combined with western medicine versus western medicine		
Wang et al. (2008) [9]	120	MD −5.56 [−7.15, −3.97]
Cui (2009) [13]	18	MD −3.23 [−9.14, 2.68]
Li (2010) [14]	48	MD −2.44 [−5.44, 0.56]
Wang et al. (2007) [15]	90	MD −3.84 [−5.35, −2.33]
Subtotal MD −4.24, 95% CI −5.59 to −2.89 *I* ^2^ = 31% fixed		
(1.2) Eye acupuncture combined with TCM herbal treatment versus TCM herbal treatment		
Liu (2010) [11]	56	MD −2.89 [−4.15, −1.63]
(1.3) Eye acupuncture combined with rehabilitation versus rehabilitation		
Jiang (2009) [20]	60	RR −2.40 [−4.87, 0.07]
(1.4) Eye acupuncture combined with rehabilitation and western medicine versus rehabilitation combined with western medicine		
Gao (2012) [22]	60	MD −2.40 [−3.76, −1.04]

(2) **Changes* *of* *ADL* *at* *the* *end of treatment**		
(2.1) Eye acupuncture combined with rehabilitation and western medicine versus rehabilitation combined with western medicine		
Chen et al. (2007) [17]	80	MD 17.60 [14.19, 21.01]
(2.2) Eye acupuncture combined with western medicine versus western medicine		
Gao (2012) [22]	60	MD 4.67 [1.45, 7.89]

(3) **Changes* *of* *SSS* *score* *at* *the* *end* *of* *treatment**		
(3.1) Eye acupuncture combined with rehabilitation and western medicine versus rehabilitation combined with western medicine		
Chen et al. (2007) [17]	80	MD 12.41 [8.92, 15.90]

(4) **Changes* *of* *FMA* *assessment* *at* *the end* *of* *treatment**		
(4.1) Eye acupuncture combined with rehabilitation and western medicine versus rehabilitation combined with western medicine		
Chen et al. (2007) [17]	80	MD 8.31 [3.15, 13.47]

(5) **Changes* *of* *HAMD score at the end of treatment**		
(5.1) Eye acupuncture combined with western medicine versus western medicine		
Huang (2013) [23]	156	MD −0.82 [−1.87, 0.23]

(6) **Changes of MMSE assessment at the end of treatment**		
(6.1) Eye acupuncture combined with western medicine versus western medicine		
Li (2009) [18]	50	MD 1.60 [0.28, 2.92]

(7) **Changes of SWT assessment at the end of treatment**		
(7.1) Eye acupuncture combined with western medicine versus western medicine		
Li and Wang (2009) [16]	100	RR 1.24 [1.03, 1.49]

(8) **Changes* *of* *NDS**		
(8.1) Eye acupuncture combined with western medicine versus western medicine		
Zhou et al. (2011) [10]	120	RR 1.08 [0.93, 1.26]

(9) **Constipation symptoms evaluation* *at the end of treatment**		
(9.1) Eye acupuncture combined with rehabilitation and western medicine versus rehabilitation combined with western medicine		
Xi (2011) [19]	60	MD −4.78 [−5.14, −4.42]

(10) **First defecation time* *at* *the end of treatment**		
(10.1) Eye acupuncture combined with rehabilitation and western medicine versus rehabilitation combined with western medicine		
Xi (2011) [19]	60	MD −1.03 [−1.46, −0.60]

(11) **Changes of* *ET* *level at the end of treatment**		
(11.1) Eye acupuncture combined with western medicine versus western medicine		
Zhou et al. (2011) [10]	120	MD −7.03 [−9.48, −5.12]
Li (2010) [14]	48	MD 68.27 [61.03, 75.51]
Subtotal 168 MD 30.40 95% CI −43.65 to 104.46 *I* ^2^ = 100% random		
(11.2) Eye acupuncture combined with rehabilitation versus rehabilitation		
Cui (2009) [13]	18	MD −10.71 [−28.09, 6.67]
(11.3) Eye acupuncture versus rehabilitation		
Xu et al. (2006) [24]	60	MD −0.64 [−1.17, −0.12]

(12) **Changes of CGRP level at the end of treatment**		
(12.1) Eye acupuncture combined with western medicine versus western medicine		
Zhou et al. (2011) [10]	120	MD 5.67 [4.03, 7.31]
Li (2010) [14]	48	MD 1.48 [−5.31, 8.27]
Subtotal 168 MD 1.48 95% CI −5.31 to 8.27 *I* ^2^ = 28% fixed		

(13) **Changes of FIB level at the end of treatment**		
(13.1) Eye acupuncture combined with western medicine versus western medicine		
Wang et al. (2008) [9]	120	MD −0.72 [−1.09, −0.35]

(14) **Changes of CRP level at the end of treatment**		
(14.1) Eye acupuncture combined with western medicine versus western medicine		
Wang et al. (2007) [15]	90	MD −5.86 [−7.54, −4.18]

(15)* * **Changes of VEGF level at the end of treatment**		
(15.1) Eye acupuncture versus western medicine		
Dong (2009) [25]	60	MD 0.02 [−0.49, 0.53]
